# 17β-estradiol induces stearoyl-CoA desaturase-1 expression in estrogen receptor-positive breast cancer cells

**DOI:** 10.1186/s12885-015-1452-1

**Published:** 2015-05-29

**Authors:** Anissa Belkaid, Sabrina R. Duguay, Rodney J. Ouellette, Marc E. Surette

**Affiliations:** 1 Department of Chemistry and Biochemistry, Université de Moncton, 18 Antonine Maillet Ave, Moncton, NB E1A 3E9 Canada; 2 Atlantic Cancer Research Institute, Moncton, NB Canada

**Keywords:** Stearoyl-CoA deasaturase-1, Estrogen, Breast carcinoma, Fatty acids

## Abstract

**Background:**

To sustain cell growth, cancer cells exhibit an altered metabolism characterized by increased lipogenesis. Stearoyl-CoA desaturase-1 (SCD-1) catalyzes the production of monounsaturated fatty acids that are essential for membrane biogenesis, and is required for cell proliferation in many cancer cell types. Although estrogen is required for the proliferation of many estrogen-sensitive breast carcinoma cells, it is also a repressor of SCD-1 expression in liver and adipose. The current study addresses this apparent paradox by investigating the impact of estrogen on SCD-1 expression in estrogen receptor-α-positive breast carcinoma cell lines.

**Methods:**

MCF-7 and T47D mammary carcinomas cells and immortalized MCF-10A mammary epithelial cells were hormone-starved then treated or not with 17β-estradiol. SCD-1 activity was assessed by measuring cellular monounsaturated/saturated fatty acid (MUFA/SFA) ratios, and SCD-1 expression was measured by qPCR, immunoblot, and immunofluorescence analyses. The role of SCD-1 in cell proliferation was measured following treatment with the SCD-1 inhibitor A959372 and following SCD-1 silencing using siRNA. The involvement of IGF-1R on SCD-1 expression was measured using the IGF-1R antagonist AG1024. The expression of SREBP-1c, a transcription factor that regulates SCD-1, was measured by qPCR, and by immunoblot analyses.

**Results:**

17β-estradiol significantly induced cell proliferation and SCD-1 activity in MCF-7 and T47D cells but not MCF-10A cells. Accordingly, 17β-estradiol significantly increased SCD-1 mRNA and protein expression in MCF-7 and T47D cells compared to untreated cells. Treatment of MCF-7 cells with 4-OH tamoxifen or siRNA silencing of estrogen receptor-α largely prevented 17β-estradiol-induced SCD-1 expression. 17β-estradiol increased SREBP-1c expression and induced the mature active 60 kDa form of SREBP-1. The selective SCD-1 inhibitor or siRNA silencing of SCD-1 blocked the 17β-estradiol-induced cell proliferation and increase in cellular MUFA/SFA ratios. IGF-1 also induced SCD-1 expression, but to a lesser extent than 17β-estradiol. The IGF-1R antagonist partially blocked 17β-estradiol-induced cell proliferation and SCD-1 expression, suggesting the impact of 17β-estradiol on SCD-1 expression is partially mediated though IGF-1R signaling.

**Conclusions:**

This study illustrates for the first time that, in contrast to hepatic and adipose tissue, estrogen induces SCD-1 expression and activity in breast carcinoma cells. These results support SCD-1 as a therapeutic target in estrogen-sensitive breast cancer.

## Background

Estrogen receptor-positive (ER + ve) breast cancer is the most diagnosed breast cancer subtype. In these estrogen sensitive cells, the role of estrogen in the maintenance and development of breast cancer is well established [1–4]. When activated by estrogen, estrogen receptors (ER) are the principal signalling molecules that regulate several oncogenic cell functions either by the genomic pathway acting directly as transcription factors in the nucleus, or by non-genomic pathways interacting with other receptors and their adjacent pathways like the insulin-like growth factor-1 receptor (IGF-1R) [5–8]. As with estrogen, it is well recognized that IGF-1/IGF-1R pathways promote cell proliferation in breast cancer cells [7, 9–11].

To sustain mitogenic growth, cancer cells are known to increase *de novo* fatty acid biosynthesis in contrast to non-malignant cells that obtain their fatty acids for membrane biogenesis from the circulation [12–14]. Effectively, in many cancers including breast cancers, acetyl-CoA carboxylase (ACC), and fatty acid synthase (FAS), the key enzymes responsible for *de novo* biosynthesis of palmitic acid, are up-regulated by the influence of oncogenic pathways unlike normal cells in which fatty acid biosynthesis is regulated through nutritional status and metabolic pathways [12, 15, 16]. Following *de novo* fatty acid biosynthesis, the enzyme stearoyl-CoA desaturase-1 (SCD-1) catalyzes the introduction of the first double bond in the *cis*-delta 9 position of saturated fatty acyl-CoA producing monounsaturated fatty acids (MUFA) that are essential for membrane biogenesis as they contribute to cell membrane fluidity [17].

Recently, SCD-1 has emerged as a potential therapeutic target since the inhibition of its activity or the silencing of its expression decreases proliferation in lung, colon, gastric, prostate, and breast cancer cell lines [18–26] and tumor formation in xenograft models [18, 24, 27]. Accordingly, SCD-1 expression is enhanced in breast and prostate cancer tissues *in situ* compared to normal tissue [26–31] and SCD-1 expression was associated with shorter survival times in breast cancer patients [27]. In both ER + ve and ER-ve breast epithelial carcinoma cell lines, mTOR inhibition reduces SCD-1 expression and cell proliferation [21] and silencing SCD-1 decreases both cell proliferation and the glycogen synthase kinase-3β-induced epithelial to mesenchymal transition [20]. Taken together, these studies demonstrate that SCD-1 expression impacts on cell proliferation and phenotype transition in an estrogen-independent manner [20, 21].

In lipogenic tissues such as the liver and adipose tissue, SCD-1 is regulated at the transcriptional level in response to nutritional status that is mediated by sterol regulatory element binding protein 1c (SREBP-1c) via a sterol response element (SRE) in the SCD-1 promoter [17, 32, 33]. Although both estrogen and SCD-1 are required for ER + ve breast cancer proliferation, paradoxically it is well documented that estrogen effectively represses SCD-1 expression in liver and adipose tissue [34–41] possibly through down regulation of SREBP-1c expression [34].

In the present study it is demonstrated for the first time that estrogen-induced cell proliferation is associated with increased SCD-1 expression and a significant increase in cellular MUFA content in ER + ve MCF-7 and T47D breast epithelial carcinoma cell lines, but not in immortalised MCF-10A breast epithelial cells. Induction of SCD-1 in ER + ve cells contradicts studies in liver and adipose tissue that report estrogen as an SCD-1 repressor [34–41]. These findings establish an important link between estrogen signaling and lipid metabolism in ER + ve breast cancer cells.

## Methods

### Reagents

Cell culture media (DMEM/F12, RPMI-1640, phenol red-free RPMI-1640), FBS, and charcoal-stripped FBS were purchased from Thermo Fisher Scientific. The IGF-1 receptor antagonist AG 1024 was purchased from EMD Millipore. The SCD-1 inhibitor A939572 was purchased from Biovision. 17β-estradiol (17β-ED), IGF-1, 4-OH tamoxifen, and DMSO were purchased from Sigma-Aldrich. 17β-ED and 4-OH tamoxifen were dissolved in ethanol, IGF-1 was prepared in sterile water and both A939572 and AG 1024 were prepared in DMSO.

### Cell culture

The MCF-7, T47D, and MCF-10A cell lines were purchased from ATCC. MCF-7 and T47D cells were maintained in RPMI 1640 medium supplemented with 10 % FBS, 100 U/ml penicillin, and 100 μg/ml streptomycin at 37 °C in a humidified 5 % CO_2_ atmosphere. MCF-10A cells were cultured as above except DMEM/F12 medium was used with 5 % FBS and 100 ng/ml cholera toxin. As described previously [42, 43], before treatments cells were cultured for one week in phenol red-free medium supplemented with 10 % charcoal-stripped FBS (5 % for MCF-10A cells) to starve cells from steroid hormones (starvation medium). Cells were then treated with 2nM 17β-ED or its vehicle in the presence of different reagents for 5 days as indicated. This concentration of 17β-ED is within the concentration range of estradiol measured in the serum of pre-menopausal women (0.2 -2nM) and of breast cancer patients (up to 3-times normal values), in breast tumors (0.25 – 2.25 pmol/g tissue) [44, 45], in the serum of mice (1.4nM) treated with estrogen pellets that promote MCF-7 tumor growth *in vivo* [46] and in the concentration range of growth promotion for cells in vitro [47]. After every experiment cells were stained with 0.2 % trypan blue and counted using a hemacytometer. In some experiments cell proliferation was assessed by flow cytometry after labeling cells with carboxyfluorescein diacetate succinimidyl ester (CFSE) using the CellTrace™ CFSE Cell Proliferation Kit as described by the manufacturer (Molecular Probes, Cat # C34554). Briefly, cells that had been starved as above for 7 days were resuspended in PBS containing 5 μM of CFSE diluted in DMSO, were incubated for 20 min at 37 °C followed by 3 washes with phenol red-free media to remove free dye remaining in the solution. The cells were then plated in starvation media with 2nM 17β-ED or its vehicle, and the media was changed every 2 days. After 5 days the cells were collected, the analyses were performed using a Beckman Coulter Cytometrics FC 500 flow cytometer and the results were analyzed with Kaluza Software.

### Transient ERα, SCD-1, and SREBP-1 siRNA silencing

Transient transfections were carried out using the Gene Pulsar X Cell from Bio Rad. MCF-7 cells (2 × 10^6^ cells) that had been starved as above for 5 days were resuspended in 200 μl phenol red-free RPMI to which was added 4 μl of siRNA targeting ERα (Cat#301461), SCD-1 (Cat # SR-304248), or SREBP-1 (Cat# SR-304579) from OriGene for a final concentration of 100nM or a non-targeting duplex of the same length as negative control (Cat # SR-30004). Cells were subjected to electroporation using a single 300 V pulse with a capacitance of 250 μF. Cells were then seeded in starvation medium (without antibiotics) containing 2nM 17β-ED or its vehicle. After 3 days, cells were collected for further analyses.

### Fatty acid analysis

Cellular lipids were extracted using a modified version of the Bligh and Dyer method [48]. Briefly, cells were detached with trypsin, washed twice with cold PBS, resuspended in 0.8 ml PBS and 3 ml of chloroform:methanol (1:2, v:v). The internal standard 1,2-diheptadecanoyl sn-glycerol-3-phosphorylcholine (3.2 μg) (Biolynx, Brockville, On), and 25 μl of 10 % acetic acid were added to each sample. Samples were vortexed and left at room temperature for 15 min. Another 2 ml of chloroform and 1 ml of water were then added, the samples were centrifuged at 180 × g for 2 min and the bottom organic layer containing lipids was transferred to a clean glass tube. Another 2 ml of chloroform was then added, and after centrifugation the bottom layer was pooled with the first extract of lipids.

The organic phase then was dried with a stream of N_2_, lipids were saponified by adding 400 μl of 0.5 M KOH in methanol and heating at 100 °C for 15 min. Fatty acid methyl esters (FAME) were then prepared by adding 500 μl of 14 % boron trifluoride (BF_3_) in methanol (Sigma-Aldrich, Oakville, On.), and heating at 100 °C for 10 min. Samples were then evaporated under a stream of N_2_, resuspended in hexane, and FAME were separated and quantified by gas chromatography (GC) using a Thermo Trace GC -equipped with a Trace-FAME column, FID detector, and Xcalibur software (Thermo, Austin TX). Peak identities, and quantities were determined by retention times and standard curves of known standards. The cellular fatty acid profiles were determined and product (16:1 n-7, 18:1n-7, and 18:1n-9) /substrate (16:0, 18:0) ratios were used as an indicator of SCD-1 activity [49].

### RNA extraction and qPCR

Cellular mRNA was extracted with Trizol (Invitrogen) and purified with the RNeasy Mini Kit (Qiagen). cDNA was prepared from mRNA using the Quantitect reverse transcription kit according to the manufacturer’s protocol (Qiagen). The efficiency of the primer pairs was evaluated using a standard curve and the stability of the expression of the RN18S1 or HPRT reference genes between treatments was evaluated. The primers for SCD-1 (137 bp) were forward- 5 ′-AGTTCTACACCTGGCTTTGG-3′ and reverse-5′-GTTGGCAATGATCAGAAAGAGC-3′, and those for SREBP-1c (164 bp) forward-5′-AGTCACTGTCTTGGTTGTTGA-3′ reverse-5′-GACCGACATCGAAGGTGAAG-3′. The primers for the reference genes are Forward 5′- GAGACTCTGGCATGCTAACTAG-3′ and reverse 5′-GGACATCTAAGGGCATCACAG-3′ and Forward 5′-TGCTGAGGATTTGGAAAGGG-3′ reverse 5′TTTATGTCCCCTGTTGACTGG-3′ for RN18S1 and HPRT, respectively. Gene expression was measured using 10 ng of cDNA by quantitative PCR (ABI 7500, Applied Biosystems) with Ssofast ^™^ Evagreen Supermix Low ROX (Bio-Rad).

### Immunocytochemistry

MCF-7 cells grown on glass cover slips at approximately 60 % confluence were then incubated in starvation medium (phenol red-free medium and charcoal-stripped FBS), followed by a 5-day treatment or not with 2nM 17β-ED as described above. Cells were fixed in 3.7 % formaldehyde for 30 minutes, permeabilized for 15 minutes with 0.1 % saponin in PBS, and incubated with 5 % non-fat dry milk in PBS for 20 minutes at room temperature. Cells were then incubated overnight at 4 °C with a mouse monoclonal anti-SCD-1 antibody from Abcam (ab19862) or an isotype control antibody. Cells were then gently rinsed with PBS and incubated with Alexa fluor-488-coupled secondary anti-mouse antibodies (Invitrogen) and 4′,6-diamidino-2-phenylindole (DAPI) for 1 hour at 37 °C. The cover slips were mounted on anti-fading mounting media (Invitrogen) and were left to dry in the dark for 24 hours. The images of fluorescent cells were taken with a digital camera and cells were visualized with an Olympus IX81 motorized inverted microscope.

### Western blot

Cells were washed in cold PBS and lysed in 50 mM Tris–HCl pH 7.6, 150nM NaCl, 2 mM EDTA and 1 % Nonidet-P40 containing a cocktail of protease inhibitors (Roche). Following a quick vortex, 5× Laemmli sample buffer (300 mM Tris–HCl pH 6.8, 10 % SDS, 50 % glycerol, 25 % β-mercaptoethanol, 0.05 % bromophenol blue) was added and samples were boiled for 10 min. Proteins were quantified by EZQ Protein Quantitation kit (Molecular probe) and cell lysates containing 50 μg of proteins were separated on 4-15 % Criterion TGX precast gels (Bio Rad). The proteins were transferred to PVDF membranes (GE Healthcare) which were then blocked in 10 % non fat dry milk in TBS-Tween. Western blotting was then performed using anti-SCD1 from Abcam (ab19862), and anti-SREBP-1 from BD Technologies (557036) that recognizes the N-terminal domain, including the mature (m) form, of SREBP-1 without distinguishing between the SREBP-1c and SREBP-1a isoforms, and horseradish peroxidase-conjugated secondary antibodies. Horseradish peroxidase-conjugated anti-B-actin was purchased from Sigma-Aldrich (A3854). The immunoblots were visualized using ECL prime (GE Healthcare) and an Alpha Innotech Fluorochem imager (San Leandro, USA).

### Statistical analyses

Data are representative of three or more independents experiments. Differences in treatments were analyzed using Student’s *t*-test or 1-way ANOVA tests with *Tukey’s* post-hoc test, performed with GraphPad Prism Version 6.0 software.

## Results and discussion

In this study we sought to investigate an apparent paradox where SCD-1 is highly expressed in breast cancers and appears to be required for appropriate cell division, although SCD-1 expression is repressed in liver and adipose tissue in response to estrogen, a principal driver of growth in ER-α-positive breast cancer. To investigate this apparent contradiction we investigated fatty acid metabolism and SCD-1 expression in response to 17β-ED treatment in ERα + breast carcinoma cell lines.

**Fig. 1:**
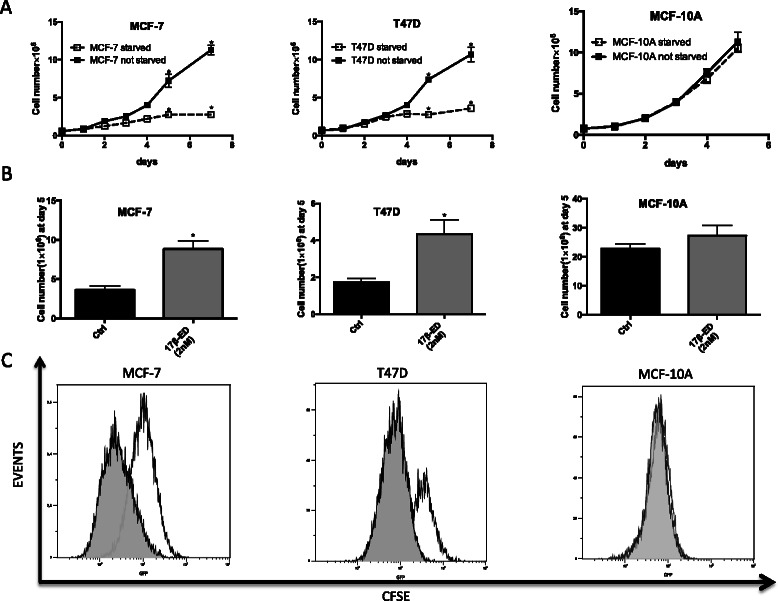
17β-estradiol induces cell proliferation in MCF-7 and T47D cells but not MCF-10A. (**a**) MCF-7, T47D, and MCF-10A cells were incubated for 7 days in phenol red-free media supplemented with charcoal-stripped FBS (starved) or with untreated FBS (not starved). The media were changed every two days and cells were counted each day. (**b**) Following one week of incubation in phenol red-free medium containing charcoal-stripped FBS, cells were treated with 2nM 17β-ED or its vehicle EtOH (Ctrl) for 5 days. The media were changed every two days and cells were then counted on day 5 post treatment. (**c**) Cells were incubated as in B above except that cells were treated with CFSE prior to incubation with 2nM 17β-ED (shaded area) or its vehicle (clear area). Cells were then assessed for CFSE content by flow cytometry on day 5 post-treatment. The result is representative of 3 independent experiments. Data are means ± SEM n = 3 independent experiments. *Different from control as determined by Student’s *t*-test (*p* < 0.05)

The cellular response to estrogen was investigated in the ERα + mammary carcinoma cell lines MCF-7 and T47D, as well as in the immortalized MCF-10A normal mammary epithelial line used as control. In this model system, when cells were starved from exogenous steroids using phenol red-free medium supplemented with charcoal-filtered serum for 7 days [42, 43, 47], MCF-7 and T47D cells ceased to proliferate compared to non-starved cells, whereas MCF-10A cell proliferation was unaffected (Fig. 1a). However, when starved cells were then incubated in the presence of 2nM 17β-ED, both MCF-7, and T47D showed a significant increase in cell proliferation as assessed by cell counting and by cellular CFSE measurement where each daughter cell retains half of the incorporated CFSE after each cell division. As expected, MCF-10A cell proliferation was unaffected by 17β-ED since these are not estrogen sensitive cells (Fig. 1b and c) [43, 50–52].

Having identified appropriate conditions in which 17β-ED induces proliferation of ERα + breast carcinoma cells, the impact of 17β-ED treatment on cellular fatty acid profiles was measured after 5 days of treatment since cell proliferation was clearly re-established at this time point. Table 1 clearly shows that cellular MUFA/SFA ratios, a measure of SCD-1 activity, increase significantly in both MCF-7 and T47D in response to 17β-ED treatment. On the other hand, MCF-10A cells show no change in fatty acid distribution following incubation with 17β-ED, a result that parallels the absence of an impact on cell proliferation in this cell line. Each of these three cell lines appears to have a particular fatty acid profile, however, the main result remains that only the cells lines in which proliferation was induced in response to 17β-ED showed an increase in MUFA/PUFA ratios and represents the first time that an ER agonist is reported to induce such an important change in cellular fatty acid profiles in ERα + breast carcinoma cells.

**Table 1:** Fatty acid composition of 17β-estradiol-treated and untreated cells

Fatty Acids	MCF-7		T47D		MCF-10A	
	Ctrl	17β-ED	Ctrl	17β-ED	Ctrl	17β-ED
16:0	19.0 ± 0.1	20.1 ± 0.4	29.6 ± 0.7	29.4 ± 1.5	20.6 ± 0.1	19.7 ± 1.4
16:1n-7	2.6 ± 0.2	9.0 ± 0.9^a^	5.9 ± 0.8	8.1 ± 0.7^a^	4.9 ± 0.1	4.4 ± 0.3
18:0	18.7 ± 0.9	13.1 ± 0.6^a^	11.6 ± 1.0	9.1 ± 0.2^a^	7.6 ± 0.1	7.5 ± 0.5
18:1n-9	22.0 ± 0.1	25.8 ± 0.4^a^	27.6 ± 0.4	27.7 ± 1.2	38.4 ± 0.2	39.9 ± 2.7
18:1n-7	4.0 ± 0.05	7.2 ± 0.8^a^	7.5 ± 0.5	9.7 ± 0.3^a^	13.4 ± 0.0	13.5 ± 5.7
Ratios	Ctrl	E	Ctr	E	Ctrl	E
16:1n-7/16:0	0.1 ± 0.0	0.4 ± 0.05^a^	0.2 ± 0.0	0.3 ± 0.0^a^	0.2 ± 0.0	0.2 ± 0.0
18:1n-9/18:0	1.2 ± 0.1	2.0 ± 0.1^a^	2.4 ± 0.2	3.1 ± 0.1^a^	5.0 ± 0.0	5.3 ± 0.0
16:1n-7 + 18:1n-7/16:0	0.3 ± 0.0	0.8 ± 0.1^a^	0.4 ± 0.0	0.6 ± 0.0^a^	0.9 ± 0.1	0.9 ± 0.2

MCF-7, T47D, and MCF-10A cells were incubated for 7 days in phenol red-free media supplemented with charcoal-stripped FBS, followed by a 5 day incubation period in the same media that was supplemented with 2nM 17β-ED or its vehicle EtOH (Ctrl)

Cellular lipids were extracted and fatty acids methyl esters were prepared and measured. Values for each fatty acid represent the percentage of total cellular fatty acids. The results are the means ± SEM, n = 3 to 5 independent experiments. ^a^Different from control (*P* < 0.05) as determined by student’s *t*-test.

**Fig. 2:**
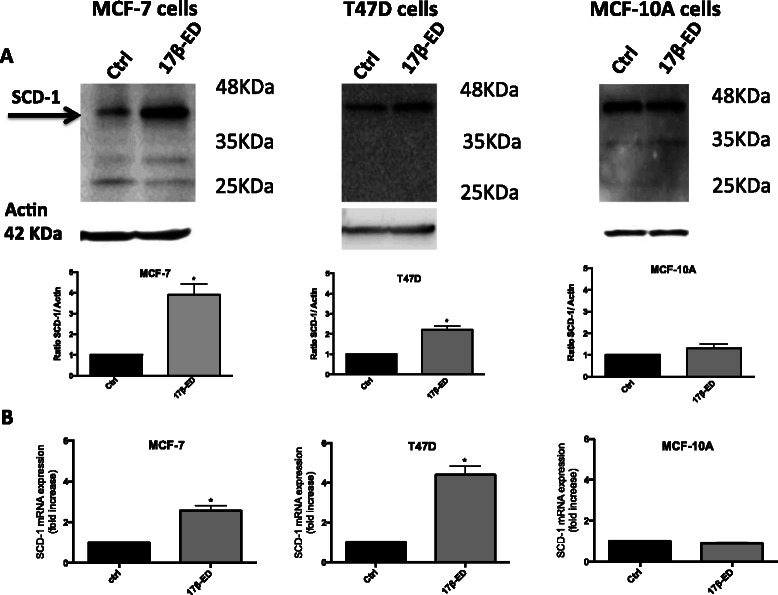
17β-estradiol increases SCD-1 expression in MCF-7 and T47D cells but not MCF-10A cells. MCF-7, T47D, and MCF-10A cells were incubated for 7 days in phenol red-free media supplemented with charcoal-stripped FBS, followed by a 5 day incubation period in the same media that was supplemented with 2nM 17β-ED or its vehicle EtOH (Ctrl). (**a**) Cellular proteins were separated by SDS-PAGE and immunoblot analysis of SCD-1 expression was performed using actin as loading control. The graphs show densitometry quantification of the SCD-1 blots. (**b**) RNA was extracted from cells and reverse transcribed into cDNA. Relative qPCR was performed using HPRT as reference gene for MCF-7 and T47D cells and RNS18S1 for MCF-10A cells. Immunoblots are representative of 3 independent experiments. Data are means ± SEM, n = 3 independent experiments. *Different from control as determined by Student’s *t*-test (*p* < 0.05)

**Fig. 3:**
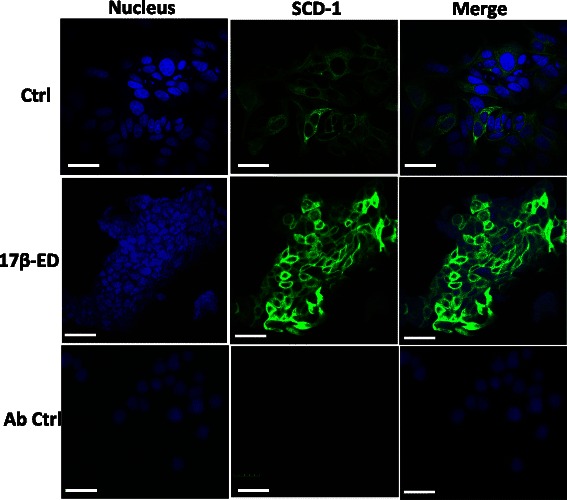
17β-estradiol increases SCD-1 levels in MCF-7 cells. MCF-7 cells were incubated for 7 days in phenol red-free media supplemented with charcoal-stripped FBS, followed by a 5 day incubation period in the same media that was supplemented with 2nM 17β-ED or its vehicle EtOH (Ctrl). Immunostaining was performed using an anti-SCD-1 antibody or its isotype control (Ab Ctrl with 17β-ED-treated cells) followed by an Alexa fluor-488-coupled secondary antibody (green) and 4–6 diamidino-2-phénylindole (DAPI) to stain nuclei (blue). Data are representative of 3 independent experiments. Scale bar = 30 μm

Since SCD-1 catalyzes the desaturation of saturated fatty acids to monounsaturated fatty acids, SCD-1 expression was measured in all three cell lines to determine whether changes in cellular fatty acid profiles were associated with an increase in SCD-1 protein expression. Fig. 2a clearly shows that 17β-ED induced the expression of SCD-1 protein in both MCF-7 and T47D cells, whereas no change in SCD-1 expression was measured in MCF-10A cells. This was accompanied by significant increases in SCD-1 mRNA content assessed by qPCR in both MCF-7 and T47D cells in response to 17β-ED, again with no measured change in MCF-10A cells (Fig. 2b). The induction of SCD-1 by 17β-ED was also apparent in MCF-7 cells when measured by immunocytochemistry (Fig. 3) and is consistent with its localization in the ER. Overall, these results are in accordance with the observed changes in fatty acid profiles of both mammary carcinoma cells lines.

**Fig. 4:**
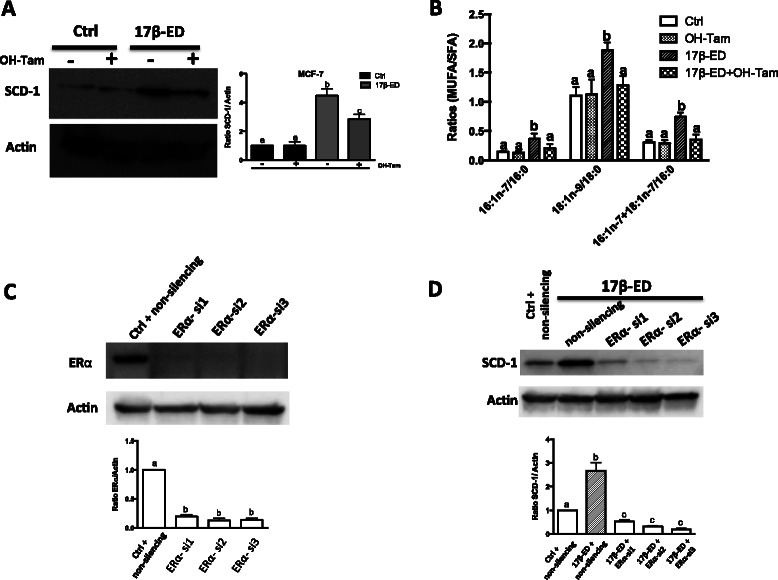
ERα silencing and treatment with 4-OH Tamoxifen blocks the 17β-estradiol induction of SCD-1 levels and activity. (**a**, **b**) MCF-7 cells were incubated for 7 days in phenol red-free media supplemented with charcoal-stripped FBS (starved), followed by a 5 day incubation period in the same media that was supplemented with 2nM 17β-ED, 10nM of 4-OH tamoxifen (OH-Tam), a combination of both, or their vehicle controls (Ctrl). (**a**) Cellular proteins were separated by SDS-PAGE and immunoblot analysis of SCD-1 expression was performed using actin as loading control. The graph shows densitometry quantification of the SCD-1 blots. (**b**) Cellular lipids were extracted, hydrolyzed, transmethylated, and quantified by GC/FID and the indicated (MUFA/SFA) ratios were calculated. (**c**) MCF-7 cells were starved for 5 days as above and were then subjected to electroporation in the presence of three different ERα-targeting siRNA (ERα-si1, ERα-si2, Erα-si3), or a non-targeting duplex (non-silencing) and were incubated in starvation medium for an additional 3 days. Cellular proteins were then separated by SDS-PAGE and immunoblot analysis of ERα was performed, using actin as loading control. The graph shows densitometry quantification of the ERα blots. (**d**) Starved cells were transfected with ERα-targeting siRNA or the non-silencing control as in (**c**) above, and were then incubated in starvation medium containing 2nM 17β-ED or its vehicle control (ctrl) for 3 days, as indicated. Cellular proteins were separated by SDS-PAGE and immunoblot analysis of SCD-1 was performed, using actin as loading control. The graph shows densitometry quantification of the SCD-1 blots. All immunoblots are representative of 3 independent experiments. Data are means ± SEM, n = 3 independent experiments. Values with a different superscript are significantly different (p < 0.05) as determined by *one-way* ANOVA test with subsequent *Tukey’s* adjustment

In order to confirm estrogen receptor involvement in the induction of SCD-1 and the changes in cellular fatty acid profiles, cells were treated with the ERα antagonist 4-OH tamoxifen or with specific siRNAs targeting ERα prior to treatment with 17β-ED. 4-OH tamoxifen significantly reduced the 17β-ED-induced SCD-1 expression and activity while ERα silencing eliminated the increase in SCD-1 expression in response to 17β-ED (Fig. 4), confirming the role of ERα in the induction of SCD-1 levels.

This induction of SCD-1 in mammary carcinoma cells in response to 17β-ED is contrary to that reported in rodents where hepatic and adipose tissue SCD-1 expression is repressed by estradiol treatment [34, 35, 38–40], and in adipose tissue from post-menopausal women treated with estradiol [37]. Similarly, 17β-ED decreases SCD-1 promoter activity in 3T3-L1 pre-adipocytes and SCD-1 expression in human hepatoma cells expressing the ERα transgene [34, 37]. The current report of SCD-1 induction in response to 17β-ED may represent a particularity of ERα + ve breast carcinoma cells that necessitate estrogen for optimal growth, which includes metabolic changes to assure a supply of unsaturated fatty acids for appropriate membrane biogenesis required to maintain a proliferative state.

**Fig. 5:**
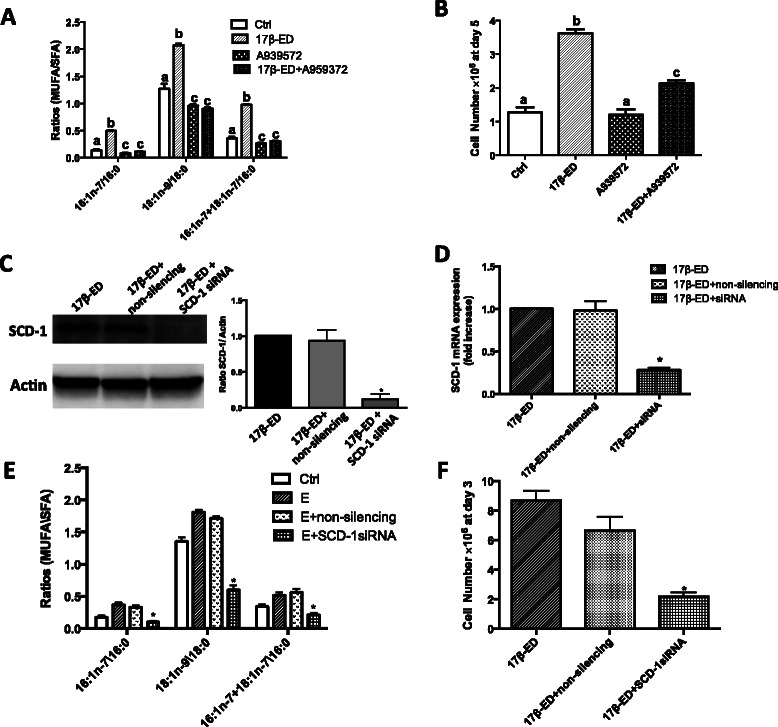
SCD-1 activity is important for 17β-estradiol induced MCF-7 cell proliferation. **(a, b)** MCF-7 cells were incubated for 7 days in phenol red-free media supplemented with charcoal-stripped FBS (starved), followed by a 5 day incubation period in the same media that was supplemented with 2nM 17β-ED, 2 μM of the SCD-1 inhibitor A939572, a combination of both, or their vehicle controls (Ctrl). (**a**) Cellular lipids were extracted, hydrolyzed, transmethylated, and quantified by GC/FID and the indicated (MUFA/SFA) ratios were calculated. (**b**) Cells subjected to the different treatments were counted using a haemocytometer. (**c**-**f**) MCF-7 cells were starved for 5 days as above and were then incubated in starvation medium containing 2nM 17β-ED for 3 days (17β-ED), or were subjected to electroporation in the presence of a SCD1-targeting siRNA (17β-ED + SCD-1 siRNA) or a non-silencing duplex control (17β-ED + NS) and then incubated in starvation medium containing 2nM 17β-ED for 3 days. (**c**) Cellular proteins were separated by SDS-PAGE and immunoblot analysis of SCD-1 expression was performed using actin as loading control. The graphs show densitometry quantification of the SCD-1 blots. (**d**) RNA was extracted from cells and reverse transcribed into cDNA. Relative qPCR was performed using HPRT as reference gene. (**e**) Cellular lipids were extracted, hydrolyzed, transmethylated, and quantified by GC/FID and the indicated (MUFA/SFA) ratios were calculated. (**f**) Cells subjected to the indicated treatments were counted using a haemocytometer. Immunoblots are representative of 3 independent experiments. Data in (**a**, **b**, **d**-**f**) are means ± SEM n = 4. Data in (**c**) are means ± SEM n = 3. Values with a different superscript are significantly different (p < 0.05) as determined by *one-way* ANOVA test with subsequent *Tukey’s* adjustment

In order to evaluate whether the 17β-ED-induced increase in SCD-1 expression and activity are required for cell proliferation, MCF-7 cells were incubated in the presence of the SCD-1 inhibitor A939572 [29, 53]. Fig. 5a shows that A939572 completely blocked the 17β-ED-induced changes in cellular fatty acid profiles with the MUFA/SFA ratios remaining nearly identical to those of control cells incubated in the absence of 17β-ED. Inhibition of SCD-1 also reversed the significant decrease in cellular 18:0 content associated with 17β-ED treatment (18.8 ± 0.6 %, 13.5 ± 0.5 % and 22.1 ± 0.9 % of cellular fatty acids for control, 17β-ED, and 17β-ED + A939572-treated cells, respectively) with no significant change in cellular 16:0 content. Importantly, treatment of MCF-7 cells with A939572 significantly suppressed 17β-ED-induced cell proliferation suggesting that SCD-1 activation is required for the induction of cell proliferation (Fig. 5b). To support the results obtained with the SCD-1 inhibitor, MCF-7 cells were also treated with siRNA targeting SCD-1. Fig. 5c and d show that the SCD-1-targeting siRNA significantly decreased SCD-1 protein and mRNA expression in 17β-ED-treated cells and this was accompanied with significant changes in cellular fatty acid composition that are consistent with the loss of SCD-1 (Fig. 5e). Importantly, siRNA silencing of SCD-1 also significantly decreased cell proliferation (Fig. 5f) confirming the results obtained with the SCD-1 inhibitor and thus confirming that 17β-ED-induced SCD-1 expression and activity are required for 17β-ED-induced cell proliferation.

In previous studies, the sensitivity of cells to SCD-1 inhibition or silencing was sometimes influenced by serum concentrations or the addition of exogenous MUFA like oleic acid. In human lung, squamous cell, colorectal, and adenocarcinoma cells lines, silencing SCD-1 resulted in growth arrest when cells were cultured in medium containing 2 % FBS [18, 29]. However, increasing the FBS content to 10 % or adding exogenous oleic acid to the cell culture medium reversed the effect of SCD-1 silencing on cell proliferation indicating that the cells could compensate for loss of SCD-1 by accessing exogenous unsaturated fatty acids. In the current study, cells were cultured in 10 % FBS that would supply ample exogenous lipids suggesting that these cells require endogenously-synthesized MUFA to support cell proliferation and that SCD-1 activity is required for ER + ve breast carcinoma cell proliferation. This is consistent with other studies reporting the inhibition of proliferation in several types of cancer cell lines, including ER + ve and ER-ve breast carcinomas, by inhibiting SCD-1 despite the presence of 10 % FBS or exogenous lipids [19–21, 25]. The reasons for this difference in reliance on SCD-1 expression and/or activity are not certain. It has been suggested that cells may accumulate saturated fatty acids (substrate) as a result of SCD-1 inhibition that causes lipotoxicity. This was supported by a synergistic effect of exogenous palmitate (16:0) with SCD-1 inhibitors on cell viability [18]. However, in the current study no increase in cellular saturated fatty acids was observed following SCD-1 inhibition or silencing, therefore differences may be related to the differential utilization of endogenous and exogenous unsaturated fatty acids for appropriate membrane biogenesis required for cell proliferation.

Previous studies have shown that SCD-1 is induced through the mTOR/eIF4E-binding protein 1 axis in breast cancer and its expression is required for mTOR-driven breast cancer cell growth [21]. SCD-1 has also been shown to be required for the modulation of signalling related to cell proliferation and epithelial to mesenchymal transition behaviour [20]. In fact SCD-1 silencing in breast cancer cells reduces ERK1/2 MAPK and GSK3 phosphorylation, and decreases β-catenin translocation to the nucleus. However, it is not clear whether SCD-1 impacts on these signalling events as a result of changes MUFA synthesis and membrane enrichment, or by a mechanism that is independent of MUFA synthesis [20]. Given that the primary cellular role of SCD-1 is to synthesize MUFA, and that the inhibition of SCD-1 activity leading to decreased MUFA production impacts on cell proliferation, it is possible that SCD-1 impacts on the above-mentioned signalling pathways by a yet-to-be-described cellular sensing mechanism for MUFA/SFA ratios.

**Fig. 6:**
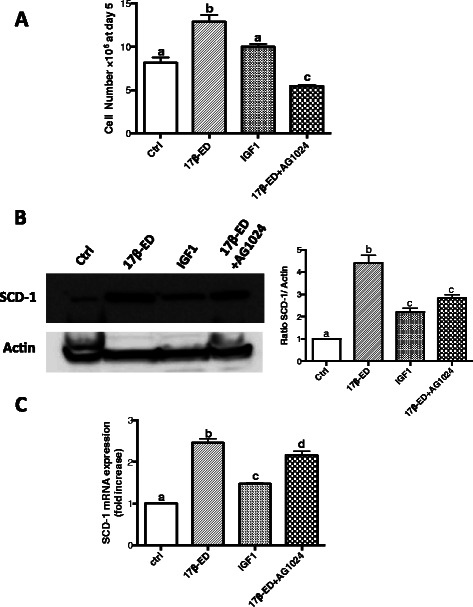
The induction of SCD-1 by 17β-estradiol partially involves IGF-1R. MCF-7 cells were incubated for 7 days in phenol red-free media supplemented with charcoal-stripped FBS (starved), followed by an incubation in the same media that was supplemented with 50 ng/ml of IGF-1, 2nM of 17β-ED, 10 μM of the IGF-1R antagonist Ag1024, a combination of 17β-ED, and Ag1024, or their vehicle controls (Ctrl) for 5 days. (**a**) Cells subjected to the different treatments were counted using a haemocytometer. (**b**) Cellular proteins were separated by SDS-PAGE and immunoblot analysis of SCD-1 levels was performed using actin as loading control. The graphs show densitometry quantification of the SCD-1 blots. (**c**) RNA was extracted from cells and reverse transcribed into cDNA. Relative qPCR for SCD-1 was performed, using HPRT as reference gene. Immunoblots are representative of 3 independent experiments. Data are means ± SEM, n = 3 or 4 independent experiments. Values that have a different superscript are significantly different (p < 0.05) as determined by *one-way* ANOVA test with subsequent *Tukey’s* adjustment

Since activation of ER-α can promote cell proliferation through cross-talk with other receptors such as the IGF1-R, it was hypothesized that activation of IGF1-R may be involved in the 17β-ED induction of SCD-1. Fig. 6a shows that the incubation of cells with IGF-1 did not induce significant cell proliferation in the absence of estrogen, but did result in an increase in SCD-1 protein expression and mRNA levels, although not as strongly as that induced by 17β-ED (Fig. 6b and c). However, treatment of cells with the IGF-1 receptor antagonist AG1024 reversed the 17β-ED-induced cell proliferation, a result consistent with the crosstalk reported between ERα and IGF-1 pathways in ERα + ve breast cancers that is associated with the promotion of cell proliferation and survival [7, 8]. Furthermore, treatment of cells with AG1024 partially prevented the 17β-ED induced induction of SCD-1 expression in MCF-7 cells suggesting that 17β-ED-induced SCD-1 expression is partially mediated through an autocrine activation of the IGF-1R. The cross talk between ER and IGF1-R is known to induce the phosphorylation and activation of MAPK and to activate the PI3K/AKT/mTOR pathway [54, 55], phenomena associated with cell proliferation. Importantly, as indicated above, mTOR activation induces SCD-1 expression in breast cancer, whereas SCD-1 expression enhances ERK1/2 MAPK activation [20, 21]. It can therefore be speculated that ER/IGF1-R crosstalk leads to the induction of SCD-1 via mTOR signaling, which in turn enables the activation of signaling cascades associated with cell proliferation.

**Fig. 7:**
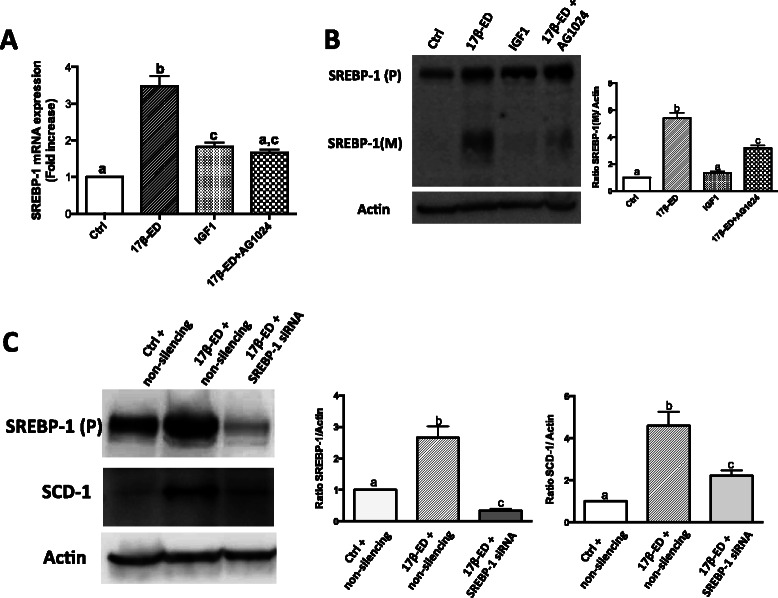
17β-estradiol induces SREBP-1C expression and activation which depend on the IGF-1 pathway. MCF-7 cells were incubated for 7 days in phenol red-free media supplemented with charcoal-stripped FBS (starved). (**a** and **b**) Starved cells were then incubated in the same media that was supplemented with 50 ng/ml of IGF-1, 2nM of 17β-ED, a combination of 17β-ED, and 10 μM of the IGF-1R antagonist Ag1024, or their vehicle controls (Ctrl) for 5 days. (**a**) RNA was extracted from cells and reverse transcribed into cDNA. Relative qPCR for SREBP-1C was performed using HPRT as reference gene. (**b**) Cellular proteins were separated by SDS-PAGE and immunoblot analysis of the precursor (P) and mature (M) SREBP-1 was performed, using actin as loading control. The graph show densitometry quantification of the SREBP-1 (M) blots. (**c**) Starved cells were then transfected with anti-SREBP-1 siRNA or its non-silencing control, and incubated with 17β-ED, or its vehicle control as indicated 3 days. Cellular proteins were separated by SDS-PAGE and immunoblot analysis of SREBP-1 (P) and SCD-1 were performed, using actin as loading control. The graphs show densitometry quantification of the SREBP-1 and SCD-1 blots. All immunoblots are representative of 3 independent experiments. Data are means ± SEM, n = 3 or 4 independent experiments. Values that have a different superscript are significantly different (p < 0.05) as determined by *one-way* ANOVA test with subsequent *Tukey’s* adjustment

SCD-1 is highly expressed in liver and adipose tissue and is primarily regulated at the transcriptional level by SREBP-1c via interaction with a sterol response element (SRE) in the SCD-1 promoter [17, 32, 33]. Accordingly, estrogen-induced repression of SCD-1 expression in liver and adipose tissue [34–37, 39, 40] has been associated with a down regulation of SREBP-1c expression [34]. Given the divergent effect of 17β-ED on SCD-1 expression in ER-α + ve breast carcinoma cells compared to liver and adipose tissues, the impact of 17β-ED on SREBP-1c expression and activation was investigated in MCF-7 cells. Unlike liver and adipose, 17β-ED increased SREBP-1c mRNA levels in MCF-7 cells (Fig. 7a). IGF-1 also induced SREBP-1c expression, though not as strongly as that measured following treatment with 17β-ED. This was accompanied with increased SREBP-1 protein expression (Fig. 7b), with 17β-ED showing a greater effect than IGF-1, similar to what was observed with SCD-1 expression. The IGF1-R antagonist AG1024 partially blocked the 17β-ED-induced expression of SREBP-1, again indicating that the effect of 17β-ED is partially mediated by crosstalk between 17β-ED and IGF-1R, possibly through an autocrine activation of IGF-1R.

The action of SREBP-1c is not only controlled at the transcriptional level since this transcription factor is activated by proteolytic cleavage of the precursor form of the protein into the mature active N-terminal form that translocates to the nucleus. Treatment of MCF-7 cells with 17β-ED resulted in the appearance of the mature form of SREBP-1 that, as with the other cellular responses, was observed to a lesser extent following incubation with IGF-1, and which was partially inhibited when 17β-ED-stimulated cells were treated with the IGF-1R antagonist (Fig. 7b). The mature N-terminal domain fragments derived from SREBP-1 detected in Fig. 7b represents the active fragment that translocates to the nucleus, but it cannot be definitively concluded that this mature SREBP-1 resulted only from SREBP-1c cleavage, since the antibody does not distinguish between the two SREBP-1 isoforms. However, silencing of SREBP-1 resulted in a significantly decreased ability of 17β-ED to induce SCD-1 indicating that 17β-ED induction of SCD-1 occurs via the SREBP-1 transcription factor (Fig. 7c).

**Fig. 8:**
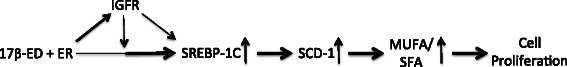
17β-estradiol-induced cell proliferation in ER-α positive breast cancer requires the activation of the transcription factor SREBP-1C which induces SCD-1 expression and a change in the cellular monounsaturated to saturated fatty acid ratio (MUFA/SFA). This induction is partially driven through the IGF-1 receptor (IGFR) and the strength of the stimulation is depicted by the thickness of the arrows. SCD-1 was previously shown to be required for the modulation of cell signalling related to cell proliferation [20]. Since SCD-1 activity is responsible for changes in cellular MUFA/SFA ratios, it is hypothesized that SCD-1 impacts on cell signalling pathways by a putative cellular sensing mechanism for MUFA/SFA ratios

Taken together, these results suggest that 17β-ED up-regulates SCD-1 expression by activating its transcription factor SREBP-1c, that this activation is partially mediated by crosstalk between ER-α and IGF-1R signaling pathways, and that the resulting change in MUFA/SFA ratios are required to support cell proliferation (Fig. 8).

## Conclusion

This study is the first to show that 17β-ED induces SCD-1 expression and the modulation of cellular lipid composition in estrogen-sensitive ER-α + ve breast carcinoma cells, and clearly demonstrates that SCD-1 expression and activity are required for estrogen-induced cell proliferation. This study also clarifies the apparent paradox where estrogen is a known repressor of SCD-1 expression in metabolic tissues, while being an activator of cell proliferation in breast carcinoma cells, a function typically associated with enhanced metabolic activity. Overall, these findings suggest that SCD-1 is a crucial player in the mitogenic effect of estrogen and supports the premise that SCD-1 is a therapeutic target in ERα + ve breast cancer.

## Abbreviations

ER: Estrogen receptor
ER + ve: Estrogen receptor-positive
CFSE: Carboxyfluorescein diacetate succinimidyl ester
SCD-1: Stearoyl-coenzyme-A desaturase
SREBP-1: Sterol response element binding protein-1
MUFA: Monounsaturated fatty acids
SFA: Saturated fatty acids
17β-ED: 17-Beta estradiol
IGF-1: Insulin-like growth factor-1
IGF-1R: Insulin-like growth factor-1 receptor
